# Extrusion and Injection Molding of Poly(3-Hydroxybutyrate-co-3-Hydroxyhexanoate) (PHBHHx): Influence of Processing Conditions on Mechanical Properties and Microstructure

**DOI:** 10.3390/polym13224012

**Published:** 2021-11-20

**Authors:** Chris Vanheusden, Pieter Samyn, Bart Goderis, Mouna Hamid, Naveen Reddy, Anitha Ethirajan, Roos Peeters, Mieke Buntinx

**Affiliations:** 1 Materials and Packaging Research & Services, Institute for Materials Research (IMO-IMOMEC), Hasselt University, Wetenschapspark 27, 3590 Diepenbeek, Belgium; chris.vanheusden@uhasselt.be (C.V.); naveen.reddy@uhasselt.be (N.R.); roos.peeters@uhasselt.be (R.P.); 2 Division IMOMEC, IMEC vzw, Wetenschapspark 1, 3590 Diepenbeek, Belgium; anitha.ethirajan@uhasselt.be; 3 Applied and Circular Chemistry, Institute for Materials Research (IMO-IMOMEC), Hasselt University, Agoralaan Gebouw D, 3590 Diepenbeek, Belgium; pieter.samyn@outlook.be; 4 Polymer Chemistry and Materials, KU Leuven, Celestijnenlaan 200F, 3001 Leuven, Belgium; bart.goderis@kuleuven.be (B.G.); mouna.hamid@kuleuven.be (M.H.); 5 Nanobiophysics and Soft Matter Interfaces Group, Institute for Materials Research (IMO-IMOMEC), Hasselt University, Wetenschapspark 1, 3590 Diepenbeek, Belgium

**Keywords:** polyhydroxyalkanoates, poly(3-hydroxybutyrate-co-3-hydroxyhexanoate, melt processing, extrusion, injection molding, mechanical properties, elongation at break, crystallization, DoE

## Abstract

Biobased and biodegradable polyhydroxyalkanoates (PHAs) have great potential as sustainable packaging materials. However, improvements in their processing and mechanical properties are necessary. In this work, the influence of melt processing conditions on the mechanical properties and microstructure of poly(3-hydroxybutyrate-co-3-hydroxyhexanoate) (PHBHHx) is examined using a full factorial design of experiments (DoE) approach. We have found that strict control over processing temperature, mold temperature, screw speed, and cooling time leads to highly increased elongation at break values, mainly under influence of higher mold temperatures at 80 °C. Increased elongation of the moldings is attributed to relaxation and decreased orientation of the polymer chains together with a homogeneous microstructure at slower cooling rates. Based on the statistically substantiated models to determine the optimal processing conditions and their effects on microstructure variation and mechanical properties of PHBHHx samples, we conclude that optimizing the processing of this biopolymer can improve the applicability of the material and extend its scope in the realm of flexible packaging applications.

## 1. Introduction

Fossil resource depletion and increased environmental awareness are driving the industry, scientific community, and general population to engage in developing and adopting more sustainable alternatives to conventional oil-based polymers [1]. Tough bioplastics currently represent only about 1% of the plastics produced annually, the market is forecasted to continuously grow to 2.87 million tons in 2025 [2]. Innovative biopolymers such as polyhydroxyalkanoates (PHAs) are one of the main drivers of growth in the field of biobased and biodegradable plastics. They are synthesized via specific bacteria and algae [3,4] from various substrates like glucose, vegetable oil, and glycerin under nutrient limitations as stress conditions [5]. Recently, more sustainable synthesis routes are using waste stream substrates from sugar, coffee, fruit, and milk production [6,7,8]. Although PHAs can be applied in a wide range of applications like packaging (e.g., films and cutlery), biomedical industry (e.g., drug carriers and tissue engineering) [9], and membrane technology (e.g., filtration) [10], challenges like high production cost, difficulties in processing and lack of clear mechanical property improvement limit their competition with conventional plastics [11].

Poly(3-hydroxybutyrate) (PHB), poly(3-hydroxybutyrate-co-3-hydroxyvalerate) (PHBV), poly(3-hydroxybutyrate-co-3-hydroxyhexanoate) (PHBHHx), and poly(3-hydroxybutyrate-co-4-hydroxybutyrate) (P3HB4HB) are PHA family members that are mostly investigated [12]. These thermoplastic polymers exhibit a wide variety of mechanical properties from hard crystalline to elastic, with medium gas permeability, depending on the co-monomer structure and content [13,14]. Co-polyesters containing PHB units with 3-hydroxyvalerate (3HV), 3-hydroxyhexanoate (3HHx), and 4-hydroxybutyrate (4HB) show reduced brittleness and better thermal stability above the melting point compared to the PHB homopolymer. PHBV and PHBHHx display slower crystallization rates than the homopolymer PHB because the co-monomer units are excluded from the PHB lattice during crystallization from the melt, which can be a challenge for efficient processing of PHBHHx, especially in methods with high cooling rates like injection molding [15,16]. Despite the rise in attempts to further improve both thermal and mechanical properties of PHAs with or without processing aids [17,18,19,20], current research regarding PHA processing and compound fabrication mostly relates to batch processing methodologies without the involvement of high temperatures and/or high shear rates, like solution casting [21,22,23], compression molding [24,25], or spinning techniques [26,27,28]. While these techniques are practical for use in a lab-scale context, they often have limited applicability on larger scales.

PHBHHx is more ductile compared to PHB and PHBV, with lower crystallinity, lower melting temperature, decreased Young’s modulus and increased elongation at break (which is highly dependent on the 3HHx content) [12,29]. However, in practice, the mechanical properties such as tensile strength, elongation at break, and Young’s modulus, together with the crystalline structures can strongly be affected by the applied melt processing method and conditions [30,31,32,33]. No studies are available to our knowledge that clearly relate the practical processing parameters in continuous melt processing like extrusion and/or injection molding of PHBHHx, with improvements in the mentioned mechanical parameters and how this, in turn, relates to the induced microstructure (orientation and crystallinity). Therefore, the current systematic and detailed study was undertaken, targeting enhanced mechanical properties of PHBHHx copolymers through optimization of the processing conditions. The obtained insights will allow for maximizing the mechanical properties and technical performance.

This paper has a twofold aim, i.e., (i) the optimization of PHBHHx melt processing targeting improved mechanical properties, including high tensile strength, high elongation at break, and—depending on the application—a low or high Young’s modulus, and (ii) the correlation of these properties with the processing-induced microstructure. A systematic design of experiments (DoE) approach is used for predictive modeling and simultaneous optimization of four extrusion and injection molding parameters: processing temperature, mold temperature, screw speed, and cooling time. The model predictions are experimentally validated in test runs, targeting a high tensile strength and high elongation at break. 

## 2. Materials and Methods

### 2.1. Materials

PHBHHx granulate (pellets) (trade name Aonilex X151A) containing 10.5 mol% 3HHx was kindly provided by Kaneka Corporation (Westerlo-Oevel, Belgium) [34]. The PHBHHx pellets were dried for at least three days at 65 °C in a circulating hot air oven prior to melt-processing to remove moisture.

### 2.2. General Sample Preparation

**Figure 1:**
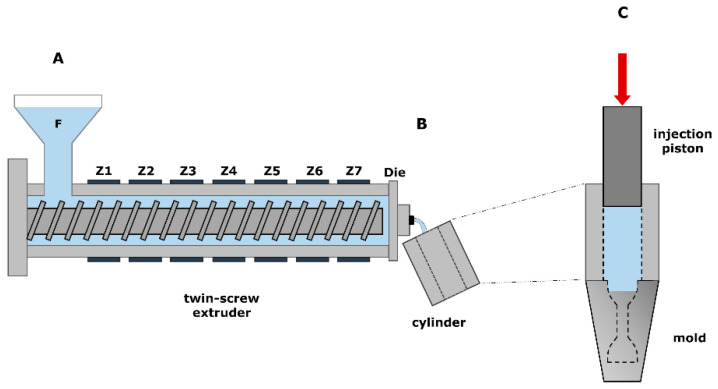
Three-step PHA melt processing with twin-screw extrusion through nine temperature zones (**A**), melt transfer into heated injection cylinder (**B**) and piston melt injection into a heated dumbbell-shaped mold (**C**).

A lab-scale co-rotating twin-screw extruder (Process 11, Thermo Scientific, Karlsruhe, Germany) and piston injection molding apparatus (Haake MiniJet II, Thermo Scientific, Karlsruhe, Germany) were used for the production of a dumbbell-shaped specimen. The dried granulate was fed through the feed hopper at a constant speed of 4 rpm into the twin-screw extruder, equipped with standard screws with a diameter of 11.0 mm and an L/D ratio of 40.0. The screw assembly includes feed screw elements (1.0 L/D), three mixing zones with kneading elements (⅟_4_ L/D), and a discharge element (1 ½ L/D) at the end (Figure 1A). A total granulate sample weight of about 10–15 g was used in order to make three replicates per processing condition. The melted polymer was immediately transferred from the extruder die-end into a heated injection cylinder (Figure 1B), which was mounted on top of a heated dumbbell-shaped mold in which the polymer was ultimately injected (Figure 1C). The injection unit pushes the polymer melt in the mold with a piston, resulting in samples of approximately 1.5 g. Afterward, the polymer samples were manually ejected from the mold and conditioned at 23 °C and 50% RH (relative humidity) for at least three days prior to characterization.

### 2.3. Full Factorial Design (FFD) Methodology

A 2^k^ full factorial design (FFD) [35] with four factors at two levels and one center-point was composed to systematically investigate the main extrusion and injection molding parameters that could influence the mechanical properties of PHBHHx. The four independent factors include: (i) the mold temperature **M**, controlled by the injection unit, (ii) the (extrusion) screw speed **S**, the speed of the two co-rotating screws, (iii) the processing temperature **P**, which relates to the extrusion die and injection cylinder temperature, and (iv) the cooling time **C**, which relates to the holding time in the mold after injection and post pressure. The injection molding parameters, such as injection pressure, injection time, post pressure, and post pressure time were held constant for comparison and to obtain consistent samples (Table 1). These parameter settings resulted from an extensive pre-screening, aiming at defining all possible combinations of upper (+) and lower limit settings (−) that led to reproducible and testable samples for analysis. Extruding at temperatures above 160 °C combined with low mold temperatures, for example, resulted in clear deterioration of the mechanical properties, difficult demolding, and defective samples. All process parameters and settings are listed in Table 1.

The final FFD includes 17 experimental runs with low (−), middle (0), and high (+) levels of each independent factor variable (Table 2). The experimental runs were executed in a random order to minimize bias. The settings of the nine temperature zones during extrusion are shown in Table 3. The extruder barrel, die, screws, and injection molding cylinder were cleaned and purged with PHBHHx between individual test runs. Each experimental run includes three repetitions (*n* = 3). 

Two specific combinations of settings of **P**, **M**, **S,** and **C** derived from modeling (A and B) were applied to produce samples for the validation testing (*n* = 5) using the confidence interval confirmation (CIC) approach [36,37].

To investigate the impact of the mold temperature in further detail, additional samples (*n* = 10) were processed at varying mold temperatures (**M** = 40, 50, 60, 70, 80 °C) with **P** = 145 °C, **S** = 50 rpm, and **C** = 60 s).

### 2.4. Predictive Modeling

The FFD was analyzed using JMP^®^ Pro Version 15 statistical software (Marlow, UK). The experimental data were used to develop predictive models that correlate melt processing parameters and mechanical properties. These empirical models contain selected first-order terms (main factors) and second-order terms (interactions) as shown in Equation (1):

(1) $$\hat{y}=\alpha_{0}+\alpha_{1}x_{1}+\alpha_{2}x_{2}+\ldots +\alpha_{12}x_{1}x_{2}+\alpha_{13}x_{1}x_{3}+\ldots$$

where $\hat{y}$ is the model response (in this work a specific mechanical property) and $\alpha_{1}$, $\alpha_{2}$, $\alpha_{12}$,… the regression coefficients. The included main factors ($x_{1}$, $x_{2}$,…) and interactions ($x_{1}x_{2}$, …) are selected if a backward stepwise regression analysis (standard least squares) indicates significance (*p* < 0.05). The actual experimental models are constructed by placing the midpoint of high (+) and low (−) factor values in the numerator of the coefficients and the difference between low/high and midpoint (0) values in the denominator, following JMP^®^ Pro software.

### 2.5. Tensile Testing

**Figure 2:**
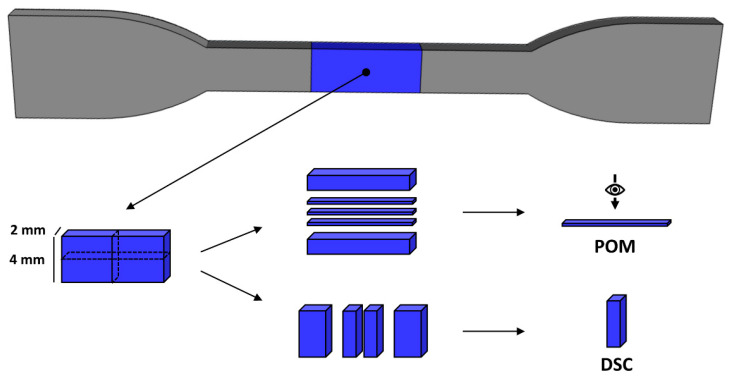
Dimensions of the dumbbell-shaped injection molded samples and cross-sections for POM and DSC analysis.

Tensile tests on injection molded samples were executed following ISO 527-2 at 23 °C and 50% RH with a 10-M systems tensile tester (MTS, Adamel Lhomargy, Roissy-en-Brie, France). Sample geometry was selected according to specimen type 5A (ISO 527-2) with a gauge length of 20 ± 0.5 mm, a width of 4.0 ± 0.1 mm, and a thickness of 2.0 ± 0.2 mm as shown in Figure 2. Tensile testing was executed with manual grips, a 2 kN load cell, a constant crosshead speed of 1 or 20 mm/min, and a grip distance of 50 mm. 

Tensile strength (TS) was calculated as the yield strength. The elongation at break (ε) used throughout this work is the nominal strain at break, which is calculated based on the grip distance and not the gauge length. The Young’s modulus (E) was determined at a crosshead speed of 20 mm/min. Tensile strength, elongation at break, and Young’s modulus were calculated with the Equations (2)–(4) respectively:

(2) $$\mathrm{TS}=\frac{F}{A}$$

(3) $$\varepsilon =\frac{L-L_{0}}{L_{0}}\times 100$$

(4) $$E=\frac{{\mathrm{FL}}_{0}}{A\Delta L}\times 100$$

where L is the final specimen length, $L_{0}$ is the initial specimen length, F is the applied force, A is the initial cross-section area, and ΔL is the change in specimen length.

### 2.6. Polarized Optical Microscopy (POM)

POM was performed on injection molded samples to investigate the semicrystalline morphology and induced microstructure. A BH-2 type microscope (Olympus, Tokyo, Japan) equipped with a ProgRes^®^ CF color camera and ProgRes^®^ Capture Pro software was used. Specimens of the narrow part of the tensile bar center were retrieved and embedded in paraffin wax for increased cutting surface and adequate clamping in the microtome apparatus. The paraffine embedded specimens were cut into 40 µm thin sections along the polymer flow direction with an RM2125 RTS manual rotary microtome (Leica, Wetzlar, Germany). The thin film samples were measured while the injection direction was at an angle of approximately 45° to the crossed polarizer and analyzer. The direction of measurement and microtome cutting of the injection molded samples are shown in Figure 2.

### 2.7. Differential Scanning Calorimetry (DSC)

PHBHHx’s melting and crystallization behavior before and after processing was evaluated using DSC measurements under inert atmosphere (50 mL/min nitrogen) using a Q200 instrument (TA Instruments, New Castle, DE, USA). Samples of about 6 mg in sealed aluminum pans were heated from −30 °C to 150 °C, before being kept isothermal for 2 min. Then, the samples were cooled to −30 °C and kept constant for 2 min before heating up to 150 °C. The heating/cooling rate was 20 °C/min. The degree of crystallinity (X_c_) was calculated using the following equation [38]:

(5) $$X_{c}=\frac{\Delta H_{m}}{\Delta H_{m0}}\times 100$$

where $\Delta H_{m}$ is the melting enthalpy of the formed crystals in the polymer and $\Delta H_{m0}$ is the melting enthalpy of the 100% crystalline polymer (115 J/g [16]). All measurements were performed in duplicate.

### 2.8. X-ray Diffraction (XRD)

The process-induced molecular orientation was assessed with XRD. Experiments were performed on a Xenocs Xeuss Mo Small Wide Angle Scattering instrument (Xenocs, Sassenage, France), comprising a GeniX 3D Molybdenum ultralow divergence X-ray beam delivery system (with wavelength λ = 0.71 Å) at a power of 50 kV−1 mA, a collimating assembly based on scatterless slits, a sample stage, a He flushed flight tube and a Mar345 image plate detector (MARresearch, Norderstedt, Germany). Injection molded samples were mounted perpendicular to the X-ray beam for the collection of 2D diffraction patterns at room temperature with an irradiation time of 30 min. 2D SAXS and WAXD data were simultaneously collected and depicted using the FIT2D software [39]. Angular calibration, azimuthal averaging and *φ* depended readouts were obtained using a *β* version of the ConeX software [40]. 

To quantify the degree of molecular orientation with respect to the injection direction (given by φ = 0), one can derive an orientation function. Orientation functions are best calculated from the *φ* dependent intensity of WAXD reflections. It is not recommended to extract quantitative orientation functions from SAXS data because in that case, crystal orientation effects cannot easily be separated from crystal size effects. Given the orthorhombic symmetry of the PHB unit cell, the normals to the 110 and 020 crystallographic planes occur at 90° with respect to the molecular chain axis. By consequence and assuming rotational symmetry around the injection axis, the molecular orientation function of chains within crystalline material can be calculated from the azimuthal evolution of the intensity of these reflections. As the foot of the 020 reflection at *φ* = 90° falls within the central blind area of the detector, it was decided to extract the molecular orientation function, *f*, from the 110 reflection according to Equation (6):

(6) $$f=1-3\langle cos^{2}\varphi_{110}\rangle$$

with

(7) $$cos^{2}\varphi_{110}=\frac{\int_{0}^{90{}^{\circ}}I{\left(\varphi \right)}_{110}sin\varphi cos^{2}\varphi d\varphi }{\int_{0}^{90{}^{\circ}}I{\left(\varphi \right)}_{110}sin\varphi d\varphi }$$

In Equation (7), $I\varphi_{110}\;$represents the intensity of the 110 reflection at a given azimuthal angle *φ* and integrated over 2*θ*. The peak integrals were obtained after separating them from the patterns using straight sectors.

### 2.9. Thermogravimetric Analysis (TGA)

TGA was performed to analyze the thermal processing window of the PHBHHx granulate using a TGA 55 (TA Instruments, New Castle, DE, USA). Samples of about 10 mg were weighted in high-temperature platinum pans and heated from 30 °C to 740 °C at a heating rate of 20 °C/min and a nitrogen gas flow of 80 mL/min.

### 2.10. Gel Permeation Chromatography (GPC)

GPC measurements were performed to determine the molar mass of PHBHHx before and after extrusion. CHCl_3_-SEC (Size Exclusion Chromatography) was performed on an HLC-8320 GPC (Tosoh EcoSEC, Griesheim, Germany) comprising an autosampler and a PSS guard column SDV (50 × 8 mm^2^), followed by two PSS SDV analytical linear M (5 μm, 300 × 8 mm^2^) columns at 35 °C and a differential refractive index detector using CHCl_3_ as the eluent with a flow rate of 1 mL/min. Toluene was used as a flow marker. The system was calibrated using linear, narrow polystyrene (PS) standards ranging from 3.70 × 10^2^–7.775 × 10^5^ g/mol PS (K = 4.9 × 10^−5^ dL/g and α = 0.794).

## 3. Results and Discussion

### 3.1. Full Factorial Design Analysis

The selection of the processing temperature **P**, mold temperature **M**, screw speed **S**, and cooling time **C** in the FFD is based on other PHA studies [33,41,42,43], preliminary DSC results, and practical considerations from an extensive pre-screening study. This selection of the processing parameters is important as mold and processing temperature are known to influence the mechanical properties of melt-processed bioplastics [44,45,46]. 

Table 4 shows a detailed summary of all executed test runs of the FFD matrix with factors and mechanical properties as a response. The average value of three sample measurements is used for calculations (*n* = 3). Large differences in elongation at break values are apparent between individual test runs. The maximum difference in elongation at break amounts to 263% elongation between individual runs nr. 1 and nr. 5. The Young’s modulus varies between 872 MPa (run 15) and 1084 MPa (run 2) and the tensile strength ranges between 20.6 MPa (run 15) and 22.4 MPa (run 2). 

**Figure 3:**
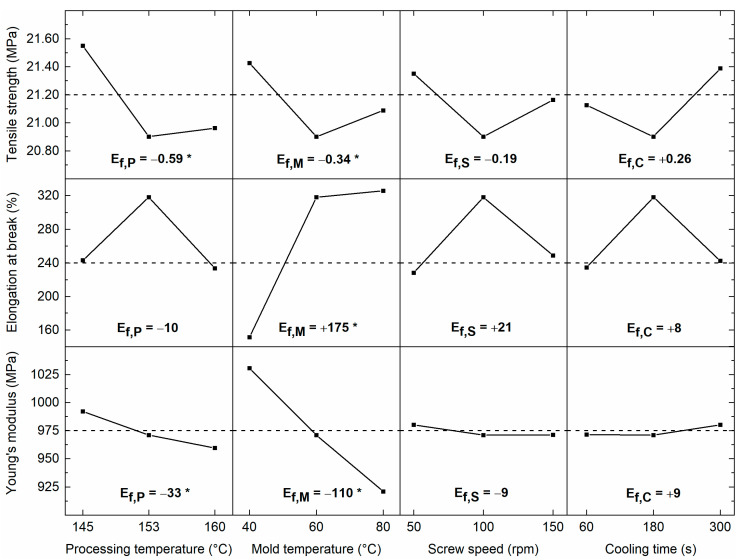
Main effects plot for tensile strength (TS), elongation at break (ε), and Young’s modulus (E) showing average values across the FFD matrix for the parameters **M**, **P**, **S,** and **C**. Significant main effects $E_{f,X}$ are indicated with an asterisk.

Figure 3 shows the main effects plots for tensile strength, elongation at break, and Young’s modulus, i.e., the mean response value at each factor level (+, 0, −) [36]. Main effects plots are a relative measure of factor significance on the system response. If the line from low to high factor levels is steep, factors have a high effect on the response. On the contrary, if the line is nearly horizontal, factors have a limited effect on the response. The sign of main effects relates to the direction of the line on the graph, showing a decrease or increase of the average response value. The center points in the main effects plot have to be interpreted with care because they are based on only one center point run (run 17). The main effects are calculated following Equation (8) [36]:

(8) $$E_{f,X}={\bar{F}}_{\left(+1\right)}-{\bar{F}}_{\left(-1\right)}$$

where $E_{f,X}$ is the main effect of parameter X on the studied response, ${\bar{F}}_{\left(+1\right)}$ is the average response at high-level factor setting, and ${\bar{F}}_{\left(-1\right)}$ is the average response at a low-level factor setting.

From Figure 3, it can be seen that processing temperature **P** has the highest influence on tensile strength. Lower processing temperatures result in higher average tensile strength values (negative main effect). The main effect of processing temperature $\left(E_{f,P}\right)$ on tensile strength equals −0.59 MPa. The effect of mold temperature **M** on tensile strength is lower and equals −0.34 MPa. The influence of screw speed **S** and cooling time **C** on tensile strength is more limited ($E_{f,S}$ = −0.19 MPa and $E_{f,C}$ = +0.26 MPa). It is also apparent from Figure 3 that mold temperature **M** has the highest impact on elongation at break. High mold temperatures result in extensively increased elongation at break values. The main effect of mold temperature on elongation at break ($E_{f,M})$ equals +175%. On the contrary, the effect of processing temperature **P**, screw speed **S,** and cooling time **C** is limited ($E_{f,P}$ = −10%, $E_{f,S}$ = +21% and $E_{f,C}$ = +8%). Further, an increased mold temperature **M** results in lower average Young’s modulus values (negative main effect). The main effect of mold temperature on Young’s modulus $\left(E_{f,M}\right)$ is −110 MPa. The effect of processing temperature **P** ($E_{f,P}$ = −33 MPa) is lower, while the effect of screw speed **S** ($E_{f,S}$ = −9 MPa) and cooling time **C** ($E_{f,C}$ = +9 MPa) are limited.

### 3.2. Effect of Processing Parameters on TS, ε, and E

Predictive models to estimate TS, ε, and E, are developed after statistical analysis in JMP^®^ Pro by selecting significant parameters and interactions (*p* < 0.05). Table 5, Table 6 and Table 7 show the terms, parameter estimates, standard error, and t-ratio of the TS, ε, and E modeling respectively if the *p*-value is below 0.05 in the backward stepwise regression method.

Table 5 shows that the main factors processing temperature **P** and mold temperature **M** have a significant effect on tensile strength. No main factor interactions are present. Optimal settings for maximum tensile strength (MPa) values are calculated with the following predictive Equation (9):

(9) $$TS=21.24-0.29\left(\frac{P-152.5}{7.5}\right)-0.17\left(\frac{M-60}{20}\right)$$

The intended goal is to increase the strength of the material. Therefore, maximized tensile strength is predicted at processing temperature **P** = 145 °C and mold temperature **M** = 40 °C and amounts to 21.70 MPa, within a 95% confidence interval (CI) of 21.44 MPa and 21.96 MPa.

Our findings of higher TS of PHBHHx samples at lower processing temperature **P** are not in total agreement with the results on PHBV processing of Vandi et al. [47]. In contrast to our results, they observed a lower TS of PHBV using a combination of low processing temperature **P** and low screw speed **S** and attributed this to an incomplete melt consolidation of the material. However, a combination of high processing temperature **P** > 190 °C and high screw speed **S** > 150 rpm also resulted in decreased tensile strength, similar to our results. This was attributed to a lower melt consolidation pressure at the extruder die due to higher melt flows when processing at higher temperatures. A decrease in PHBV mechanical performance at higher process temperatures was also reported elsewhere [33].

An explanation for a decrease in tensile strength at elevated process temperatures could be found in the fact that PHBHHx twin-screw extrusion and injection molding at high temperatures can lead to a reduction in molecular weight due to thermal degradation [18]. Random chain scission has been reported as the degradation mechanism causing a rapid decrease in molecular weight of PHAs during thermal treatment [46]. The theoretical processing window of the PHBHHx granulate is determined as the temperature region between the melting temperature of 130.5 °C (DSC) and the onset degradation temperature of 279.5 °C (TGA) and should be indicative for suitable melt processing of the polymer. Processing at temperatures of **P** = 145–160 °C seems suitable with a granulate peak degradation temperature of 295.3 °C (determined with TGA, and similar to the literature [48]). Although the processing temperature **P** was chosen well in the lower part of this theoretical range, the practical processing window is smaller because thermo-mechanical and thermo-oxidative degradation of the polymer might occur due to high shear (high rotational screw speed) at elevated temperatures [49]. 

Thermal degradation was quantified in separate GPC (SEC) experiments as the loss in molecular weight after processing at different temperatures. The weight average molecular weight (M_w_) and number average molecular weight (M_n_) of the unprocessed pellet and samples processed at different temperatures are shown in Table 8. The M_w_ after processing at 145 °C, 160 °C, 170 °C, and 180 °C decreases respectively with 13%, 23%, 19%, and 48%; the M_n_ does not decrease at **P** = 145 °C but decreases with 3, 8 and 37% at **P** = 160 °C, 170 °C, and 180 °C, respectively. This reduction in molecular weight might explain the decrease in TS when the temperature is increased (Figure 3). Processing at 180 °C seems not suitable due to a severe decrease in the polymer molecular weight. A decrease of molecular weight of PHB and PHBV (in the range of 4–53%) at elevated temperatures was reported earlier [50] and can decrease the tensile strength [51]. Decreased tensile strength of PLA by reduction of molecular weight was partially attributed to a decrease in chain length and number of chain entanglements [52]. Molecular weight decrease after processing at elevated temperatures was also reported for conventional PS and PMMA [53].

Concerning the elongation at break modeling shown in Table 6, the statistical analysis indicates that only the mold temperature **M** has a significant effect on this property. No main factor interactions are present. Optimal settings for maximum elongation (%) values are calculated with the following predictive Equation (10):

(10) $$\varepsilon =243.06+87.25\left(\frac{M-60}{20}\right)$$

The intended goal is to increase the elongation of the material. Therefore, maximized elongation is predicted at mold temperature **M** = 80 °C and amounts to 330%, within a 95% CI of 301% and 360%.

Young’s modulus is significantly affected by the main factors mold temperature **M** and processing temperature **P** (Table 7). No main factor interactions are apparent. Young’s modulus values (MPa) are calculated with the following predictive Equation (11):

(11) $$E=975.41-16.31\left(\frac{P-152.5}{7.5}\right)-54.81\left(\frac{M-60}{20}\right)$$

The intended Young’s modulus value of the material is dependent on the application. Therefore, maximized Young’s modulus is predicted at 145 °C processing temperature **P** and 40 °C mold temperature **M**, amounting to 1047 MPa (95% CI: 1026–1067 MPa). On the contrary, minimized Young’s modulus is predicted at 160 °C processing temperature **P** and 80 °C mold temperature **M**, amounting to 904 MPa (95% CI: 884–925 MPa).

### 3.3. Predictive Model Validation

The mathematical models described in the previous section highlight the selection of significant processing parameters and their effect on the separate mechanical properties. By combining these models, optimal PHBHHx processing conditions to maximize the elongation at break (while maintaining high tensile strength) are selected as: processing temperature **P** = 145 °C, mold temperature **M** = 80 °C, screw speed **S** = 50 rpm, and cooling time **C** = 60 s (settings A). An optimal mold temperature **M** of 80 °C is selected because the effect of a low mold temperature **M** = 40 °C on tensile strength is limited. Since long(er) cooling times of samples in the mold (300 s) lead to increased injection molding cycle times and lower productivity, an optimal cooling time **C** of 60 s is selected because of the influence of this parameter on mechanical properties—especially elongation at break—is limited. According to the model and for comparison, processing temperature **P** = 160 °C, mold temperature **M** = 40 °C, screw speed **S** = 50 rpm, and cooling time **C** = 60 s, are considered as non-optimal processing conditions resulting in lower elongation at break and tensile strength (settings B), while minimizing the injection molding cycle time. 

The goal of the FFD and model development is to predict optimal processing conditions for maximized mechanical properties (i.e., the intended use), not to predict the total design space in detail. Therefore, only a validation of the models for both predicted settings A and B is performed. The validation of the predictive models is executed following the confidence interval confirmation approach (CICon). This approach includes the calculation of confidence intervals using the confirmation runs themselves, rather than the confidence intervals of the experimental runs [36,37]. If the predicted values of the mechanical properties fall within this confidence interval (CI), the model is considered appropriate. The predicted mechanical properties are validated at a tensile test speed of 20 mm/min for both melt processing settings and are shown in Table 9. A 95% CI is used to assess the prediction quality of the model. 

The predictive models show good estimations for elongation at break and Young’s modulus. For tensile strength, however, they show a slight underestimation for both test settings. The underestimations can be attributed to very small deviations of tensile strength in function of processing conditions, resulting in relatively small confidence intervals. Higher prediction accuracy for tensile strength can be achieved by adding repetitions, replications, or extra points to the design. 

In addition, samples from both validation runs are also compared at a tensile test speed of 1 mm/min. As the model is built on tensile properties measured at 20 mm/min, no prediction values for 1 mm/min are available in Table 9. Tensile testing at a lower speed (1 mm/min) shows similar results for Young’s modulus at a test speed of 20 mm/min. However, testing the PHBHHx samples at low speed shows lower values of tensile strength and elongation at break compared to higher speeds.

Referring to the first objective, this study shows that PHBHHx can be melt-processed in products with TS ranging between 20–22 MPa, ε ranging between 19–342%, and E ranging between 883–1205 MPa depending on the processing conditions and tensile test method. The tensile strength of PHBHHx is relatively low compared to semi-crystalline PLA (50–70 MPa) [54], isotactic PP (29–39 MPa), PHB (40 MPa) [12], PHBV (30–38 MPa) [12] and LDPE (15–79 MPa) [12]. The elongation at break of PHBHHx is high compared to semi-crystalline PLA (4%) [54], PHBV (0.8–58%) [55,56,57] and PHB (3–8%) [12], and closer to ε values found for isotactic PP (500–900%) [13] and LDPE (150–600%) [12]. The Young’s modulus of PHBHHx is higher compared to LDPE (50–100 MPa) and in line with values found for isotactic PP (1000–1700 MPa) [12], but lower as compared to semi-crystalline PLA (3000 MPa) [54], PHBV (700–2900 MPa) [12] and PHB (3500–4000) [12]. In conclusion, the flexibility of PHBHHx can be adapted within the range of flexible packaging materials by an appropriate selection of processing conditions.

### 3.4. Influence of the Mold Temperature on Mechanical Properties

**Figure 4:**
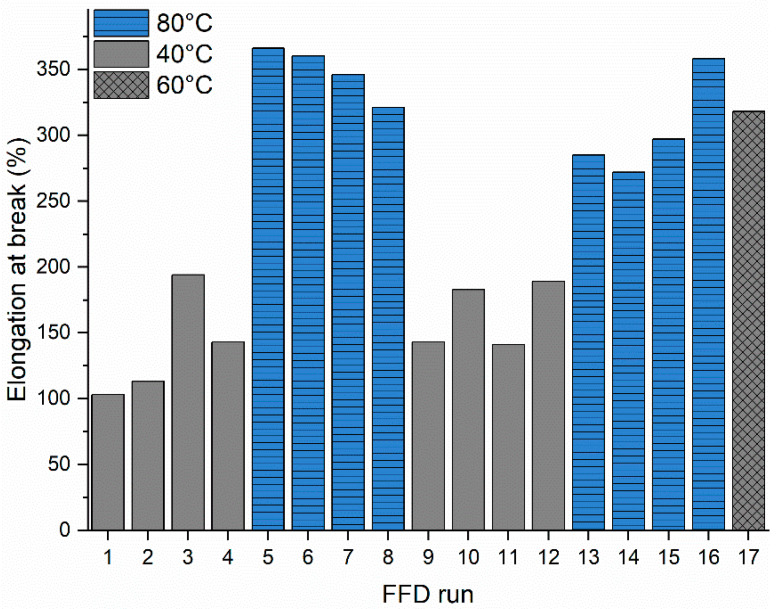
Individual value plot of the 17 run FFD for elongation at break (ε), grouped according to mold temperature **M** = 40 °C (grey), **M** = 80 °C (blue), and **M** = 60 °C center point run (grey, cross) showing increased elongation at break for high mold temperatures (only mean values are represented for clarity, statistical error is included in Table 4).

The FFD and developed prediction models revealed that the mold temperature **M** has a significant impact on the mechanical properties of the melt-processed PHBHHx (Table 4). Figure 4 graphically shows that the elongation at break of injection molded samples significantly increases with mold temperature **M** up to 80 °C and deteriorates at low mold temperature **M** = 40 °C. 

**Figure 5:**
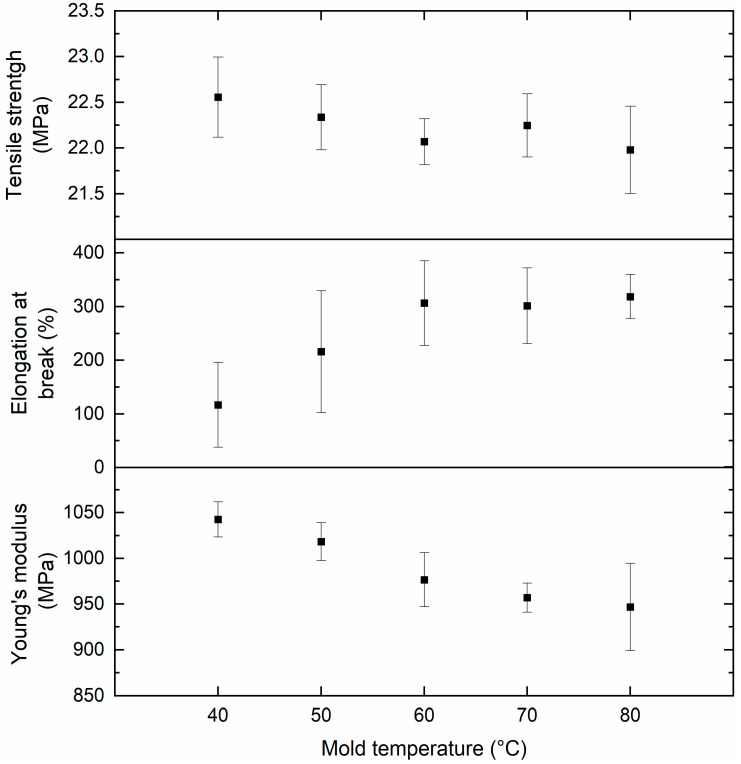
Correlation of mold temperature **M** and mean mechanical properties: tensile strength, elongation at break, and Young’s modulus (*n* = 10, ± 1 SD).

In order to investigate the underlying causes and to fully characterize mold temperature dependence, additional PHBHHx samples were produced at mold temperatures **M** = 40 °C, 50 °C, 60 °C, 70 °C and 80 °C (**P** = 145 °C, **S** = 50 rpm, **C** = 60 s). The mechanical properties were characterized by tensile testing at 20 mm/min. Figure 5 provides the experimental data for TS, ε, and E in relation to the mold temperature **M**. A clear trend of increasing elongation at break with mold temperature is observed, with stagnation at 60–70 °C. On the contrary, both the tensile strength and Young’s modulus decrease with increasing mold temperature. Similarly, it is apparent from the FFD matrix (Table 4) that ε values increase on average from 103% when molding at 40 °C, to 366% when molding at 80 °C. In addition, there is less variation in ε for samples molded at 80 °C, which can be an indication of consistent microstructure development. The combination of increased elongation and decreased Young’s modulus at higher mold temperatures shows that PHBHHx is rendered more ductile and less stiff when processed at optimal conditions (A) applying a higher mold temperature.

### 3.5. Influence of the Mold Temperature on Process-Induced Microstructure

The thermal and mechanical behavior of PHBHHx is strongly dependent on the crystal structure [29,32]. In order to investigate the observed impact of the mold temperature **M**, on TS, ε, and E properties, DSC, POM, and XRD measurements are performed to characterize the developed crystallinity and microstructure during processing. 

**Figure 6:**
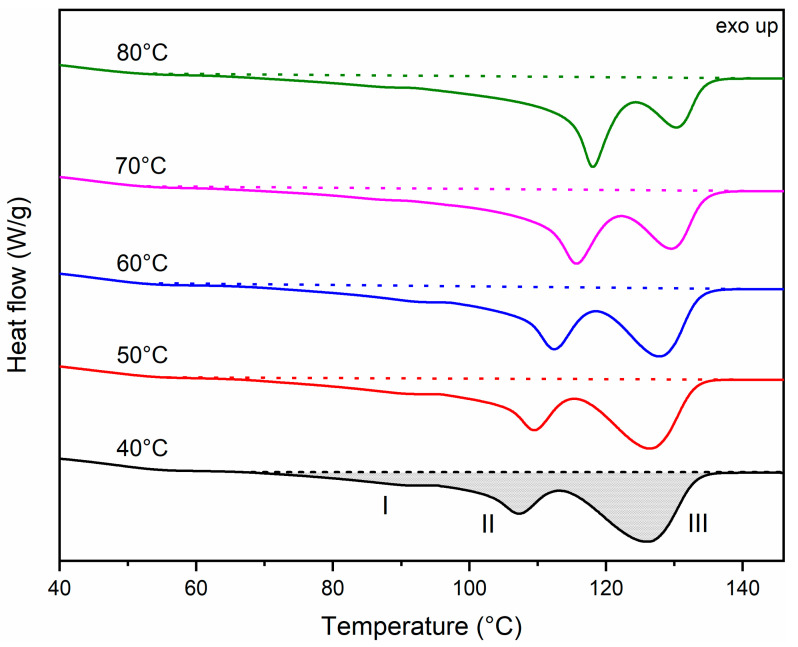
DSC first heating curves of PHBHHx samples with variable mold temperatures **M** = 40 to 80 °C with three endothermal peaks (I–III) for crystallinity calculations.

The DSC first heating curves of samples molded at temperatures of **M** = 40–80 °C are shown in Figure 6. The glass transition temperature (T_g_) of all samples equals −1 to 1 °C (similar to the literature [48,58]). As no shift is observed, T_g_ is not shown in Figure 6. Three endothermal peaks are apparent in this first heating curve. The small peak I at ±84–89 °C can be attributed to the melting of secondary crystallites formed during secondary crystallization/molding. Peak II at ±107–119 °C is due to the melting of primary lamellae [16,59]. Peak III is due to the melting of lamellae formed through thickening and reorganization during DSC heating [16,32]. It is apparent from Figure 6 that the melting peak temperature of the primary lamellae (peak II) shifts to higher temperatures and the peak area increases when processed at elevated mold temperatures (less recrystallization during DSC). Allowing time to crystallize at higher temperatures in the mold leads to increased crystal sizes with higher melting points. In addition, the area of peak III decreases with increasing mold temperature, indicating a less exhaustive recrystallization process during heating. All peak temperatures are shown in Table 10.

The calculation of the processing-induced crystalline content is based on the sum of enthalpies (ΔH_m_) of the three endothermic peaks. These peaks hide the exothermic heat due to recrystallization, which is expected to be as large as the endothermic melting enthalpy of the recrystallized crystals. As a result, the full integral actually corresponds to the melting enthalpy of the processing induced primary and secondary crystals. The crystallinity remains constant with increasing mold temperature **M**, around ±38–39% (Table 10). These results differ from a study on PLA, where almost no crystallization was observed after molding at room temperature or up to 50 °C [60,61]. Crystal formation and enlargement in PLA was achieved by post-annealing at temperatures of 80–120 °C, with a maximal increase of elongation at break when annealing occurred at 80 °C for 0.5 and 2 h [61]. As the mold temperature **M** has no effect on the crystallinity of the injection molded PHBHHx, other differences in microstructure, orientation effects, in particular, are investigated hereafter to account for the observed variations in mechanical properties.

Several studies have shown that melt processing parameters like mold temperature **M** and processing temperature **P** can induce order of magnitude variations in mechanical properties like elongation at break, due to microlayer development in the moldings [62,63,64]. A conventional three-layer process-induced morphology has been reported for a range of materials (PP, polyamide, poly(butylene terephthalate)…), with different semi-crystalline characteristics between the skin, shear, and core layers [65,66,67]. By increasing the mold temperature, the shear and skin layers reduce in thickness, providing more volume to the spherulitic core layer [68,69,70]. Morphological analysis on PHBHHx samples produced at mold temperatures **M** from 40 °C to 80 °C was performed in order to investigate the process-induced microstructure. 

**Figure 7:**
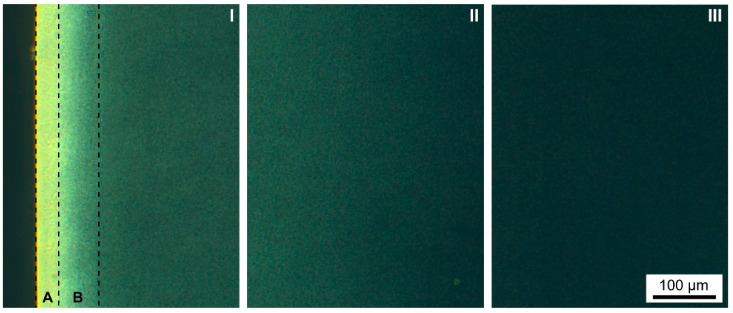
Optical analysis. POM micrographs on 40 µm thin sections with mold temperature **M** = 40 °C showing birefringence gradient existing in the different regions from the skin to the core (**I**–**III**). (**A**) and (**B**) represent the skin and shear-like layers, respectively.

POM micrographs representing outer (I), middle (II), and inner (III) regions of a sample produced at **M** = 40 °C are shown in Figure 7. It can be seen that a distinctive skin-core layer structure is apparent, with high birefringence in the skin region and a gradual brightness decrease towards the core. The skin birefringence is an indication of molecular chain orientation [71]. The birefringence is absent towards the core because the polymer chains are randomly oriented, becoming optically isotropic [71]. The skin and shear layer are defined as regions A and B in Figure 7 based on birefringence intensity. The thickness of the skin (A) and shear layer (B) are respectively ±35 µm (±1.75% of sample thickness) and ±65 µm (±3.25% of sample thickness) on both sides of the sample. Hence, the total skin-like region is approximately 10% of the sample thickness. No spherulites are visible in the core region (no Maltese crosses) because it might be that the thin films (40 µm) are thicker than the spherulite size.

**Figure 8:**
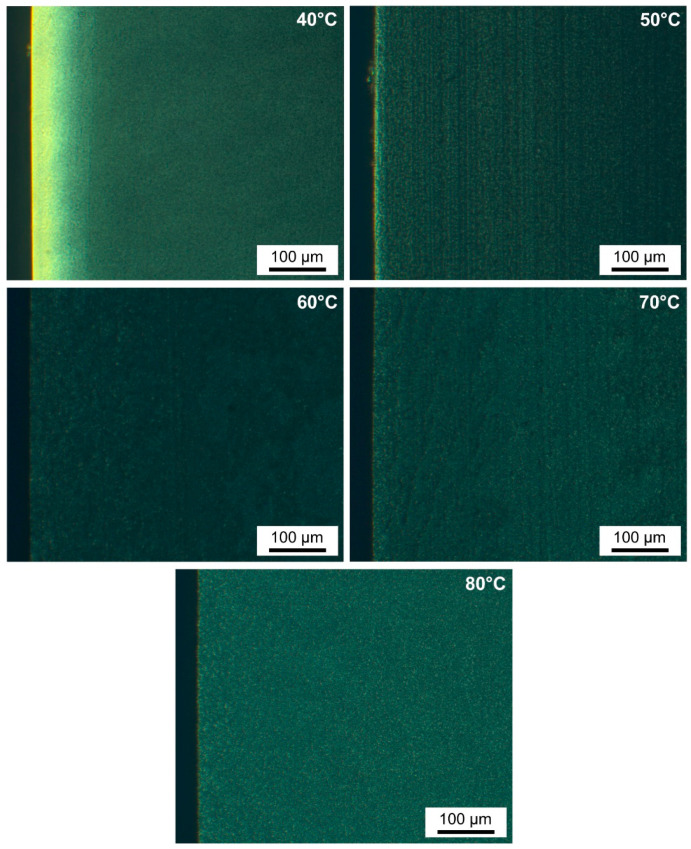
POM micrographs with 20× zoom on 40 µm thin sections for mold temperatures **M** ranging from 40–80 °C.

POM of samples produced at mold temperature **M** = 50 °C (Figure 8), shows a decrease in the thickness of the skin layer to ±10 µm (±0.50% of sample thickness) on both sides of the sample, without a distinguishable transition layer towards the core. The skin-like region is reduced from 10% at **M** = 40 °C to approximately 1% at **M** = 50 °C, with respect to the total sample thickness. The other POM micrographs in Figure 8 show that skin formation is absent at higher mold temperatures of **M** = 60, 70, and 80 °C. The oriented polymer chains are relaxed at elevated mold temperatures prior to solidification, which reduces orientational birefringence [71]. Hence, these moldings are characterized by a homogeneous and less oriented microstructure throughout the whole sample. At **M** = 80 °C, spherulites are visible because their size is increased compared to samples at **M** = 40 °C. 

**Figure 9:**
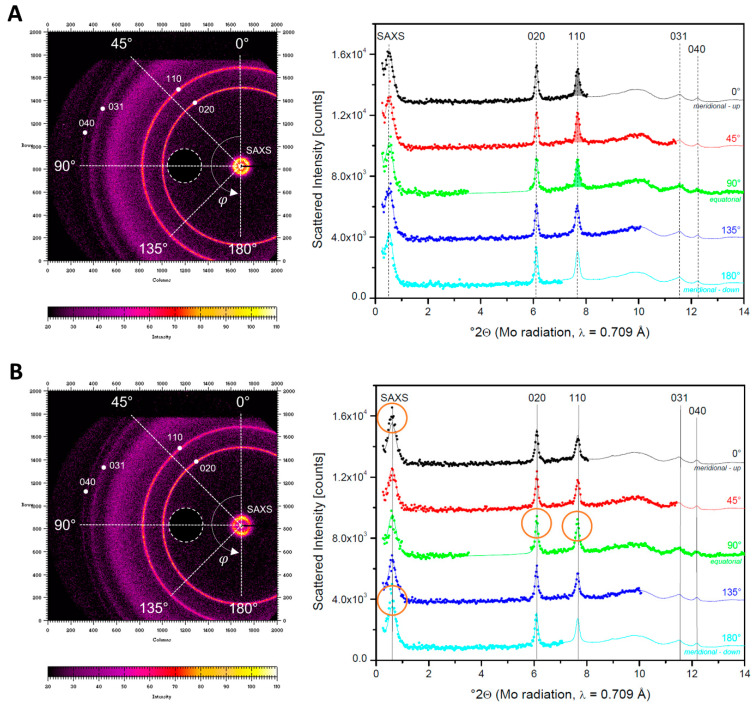
2D scattering patterns of samples **M** = 80 °C (**A**) and **M** = 40 °C (**B**) with an indication of the PHB typical reflections with Miller indices (**left**) and corresponding individual scans along different *φ* directions (**right**). The white circled regions in the 2D scattering patterns are detector blind regions.

XRD data were obtained in transmission through the central part of the tensile test bar. As a result, these patterns are superpositions of the scattering coming from the bulk as well as the surface of the samples. The 2D scattering patterns in Figure 9 (left side) of **M** = 40 °C and 80 °C show typical PHB reflections with Miller indices 020, 110, 031, and 040 [72]. The individual scattering angle (2θ) scans along *φ* directions of 0°, 45°, 90°, 135°, and 180° (Figure 9, right side) are compared to the azimuthally averaged patterns (full lines) obtained by averaging the intensities over al *φ* directions. The injection molding flow direction is parallel to the meridional direction (*φ* = 0°).

For **M** = 80 °C (Figure 9A), the scans over the different *φ* directions yield intensities equal to those of the azimuthal average. This means that no orientation is apparent.

For **M** = 40 °C (Figure 9B), the SAXS peaks are shifted to larger scattering angles compared to **M** = 80 °C, implying that the crystal-to-crystal separation for **M** = 40 °C is shorter. As the (DSC based) crystallinity for **M** = 40 °C is equal to that of **M** = 80 °C, one can conclude that the crystals for **M** = 40 °C, are thinner. No efforts were made to further quantify the size differences. The reflections in the WAXD part have not shifted drastically and peak intensities are comparable, indicative of a similar crystallinity as in samples with **M** = 80 °C and confirming the presented DSC data. The circled intensities of the angular scans (Figure 9B, right) are larger than the azimuthal average and point at (partial) molecular orientation along the injection axis (*φ* = 0°). It can be seen that the 020 and 110 reflections are stronger along the equator, meaning that a fraction of the chains (logically residing in the skin layer) are oriented parallel to the injection molding direction. However, the crystalline lamellar stacks are oriented perpendicular to this direction because the SAXS intensity is stronger along the meridional. This scattering behavior is typical of shish kebab structures as, e.g., observed earlier for polyethylene, which also has an orthorhombic crystal structure [73]. The orthogonal relation between the WAXD and SAXS reflections furthermore indicates that the chains within the crystals are oriented perpendicular to the lamellar surface direction.

It can qualitatively be derived from the scattering pattern that the orientation for **M** = 40 °C is not very large. This corresponds well with the POM finding that orientation is limited to the skin. The scattering from the core is isotropic, comparable to **M** = 80 °C. To quantify the degree of molecular orientation with respect to the injection direction (given by *φ* = 0), orientation functions f were derived from the *φ* dependent intensity of the 110 reflection. Intensities are obtained by integrating the 110 peaks after separating them from the patterns using straight sectors. To illustrate the integration procedure, the areas of the 110 reflections for φ equal to 0, 45, and 90 °C, are color shaded in Figure 9A. The values of the orientation function f in relation to mold temperature **M**, tensile properties and crystallinity are shown in Table 11. Zero values for f are obtained when the crystals and the chains they contain are oriented randomly and values of 1 correspond to perfect alignment with respect to the injection melt flow direction [74]. The molecular orientation is significant for **M** = 40 °C and 50 °C, but very small for the other mold temperatures **M** = 60–80 °C. The as-obtained molecular orientation parameter f only relates to the crystalline parts of the material. The overall molecular orientation is presumably less than given by f because amorphous matter more readily randomizes. This difference may be more important for **M** = 50 °C compared to **M** = 40 °C, as the POM experiments clearly indicate that the overall orientation in the skin is less developed for **M** = 50 °C. The increased molecular orientation at low mold temperature occurs due to polymer chain molecules being aligned, sheared, and stretched in the direction of flow during the injection. The frozen orientation is more retained at faster cooling rates of the polymer melt [75], i.e., at lower mold temperatures. The orientation effect is highly pronounced in polymer regions near the mold wall, where molecules are frozen in their stretched state, forming a skin-like layer. Taken together, the XRD and POM results suggest that processing at low mold temperature (**M** = 40 °C) induces skin formation and increased molecular orientation in the sample, while the latter is minimized at higher mold temperatures (**M** = 80 °C), i.e., at slow cooling rates.

The results of the microstructural analysis from DSC, POM, and XRD measurements indicate that the mold temperature changes the crystallization and orientation conditions of the melt during processing. Molding at low mold temperature (40 °C) produces small crystals with high molecular orientation in a skin-like structure comprising approximately 10% of the sample thickness. Due to the stretching and shearing of the polymer chains during injection molding, the inherent maximum stretch and strength of the chains are nearly reached compared to their initial relaxed state [75]. This orientation results in slightly increased tensile strength and Young’s modulus but highly reduced elongation at break. On the contrary, the selected optimal processing condition (A) with higher mold temperature (80 °C) gives rise to slower cooling and a suitable time interval for the polymer chains to relax before solidification by crystallization, compared to molding at a lower temperature. Processing of PHBHHx at higher mold temperature reduces process-induced skin formation and molecular orientation. The produced samples have increased crystal size (derived from DSC and qualitatively from SAXS) and a homogeneous microstructure throughout the sample thickness (derived from POM). This microstructure results in increased elongation at break values because the randomly curled and oriented polymer chains can be stretched to a further extent [76]. In addition, increased elongation values at **M** = 80 °C are also likely to be related to lower residual stress of the polymer chains induced by slower cooling rates of the melt during molding [71,77]. 

In this study, a higher mold temperature was found to cause improved elongation at break due to a reduction of molecular orientation and skin formation. The increased elongation at break was not attributed to changes in the overall crystallinity. This finding is contrary to previous studies which have suggested that higher mold temperatures result in lower elongation at break, mainly due to the formation of larger spherulites and increased crystallinity. Fast cooling (quenching) of the PHB melt is related to the formation of smaller crystallites, while slow cooling results in larger spherulites [31]. These large spherulites are more brittle, giving rise to poor mechanical properties like short elongation and low impact strength. Increased crystallinity at elevated mold temperatures was reported for PLA [45] and isotactic PP [78,79], leading to decreased elongation at break and impact properties. This negative effect on mechanical properties was attributed to a volumetric increase of more brittle/rigid crystalline regions and a decrease of loosely arranged amorphous regions, causing brittle instead of ductile failure. 

Several other attempts to improve the mechanical properties of PHAs were made previously by incorporating for example fillers and plasticizers. The addition of plasticizers in PHB can improve the elongation at break with values up to 45% but at the expense of tensile strength and Young’s modulus [17]. Some talc-filled PHBHHx compounds remain very brittle with elongation values under ±5% [18], while the addition of *L*-phenylalanine [19] and ultrafine talc [20] nucleating agents have almost no effect on elongation at break. The limited increase of mechanical properties, i.e., elongation at break for PHAs in these studies highlights the importance of process optimization, as discovered in this work. As shown here, elongation at break values can be increased extensively with average values up to ±175% by optimizing process conditions. However, increasing the crystallization rate and nucleation by incorporation of fillers and the combination of efficient and optimal processing conditions can possibly improve and modify the mechanical properties of PHBHHx to a further extent. In addition, PHBHHx post-annealing can be a valuable approach to promote crystallization and improve mechanical properties, as previously shown for PLA [80].

## 4. Conclusions

In this study, the influence of melt processing parameters (processing temperature **P**, mold temperature **M**, screw speed **S,** and cooling time **C**) on the mechanical properties and microstructure of the injection molded PHBHHx was systematically investigated by a full factorial design of experiments approach. Increased tensile strength and elongation at break values are found when PHBHHx is processed at relatively low extrusion and injection molding temperature profiles of 140 to 145 °C (**P**), relatively high mold temperatures of 80 °C (**M**), low screw speeds of 50 rpm (**S**) and short cooling times in the mold of 60 s (**C**). Increased cooling in the mold of 300 s gives rise to slightly increased tensile strength but is not practical for use in industry. The statistically developed empirical models predict an optimal elongation at a break value of 330% at optimized processing conditions (A) with high mold temperatures compared to 156% at non-optimal processing conditions (B) with low mold temperatures.

This extensive increase in elongation at break of PHBHHx moldings at optimal processing conditions is mainly attributed to high mold temperatures, retarding the cooling in the mold. This results in a suitable time interval to allow chain relaxation prior to crystallization-induced solidification. An increase in the mold temperature does not change the sample crystallinity content under the optimized processing conditions. In contrast, molding at a lower temperature (40 °C) induces partial polymer orientation and skin-core formation, resulting in lower elongation values.

This study highlights the importance of optimal melt processing and the influence on both mechanical properties and developed microstructure. Further research includes the combination of process optimization, incorporation of fillers, and post-annealing to further improve the mechanical properties of these PHA materials. This knowledge can contribute to the development of innovative PHA materials for packaging and other applications, taking into account the importance of the relation between melt process parameters and the possibility to enhance the mechanical properties of PHBHHx by process optimization. Our results could be used as an initial guideline for appropriate processing at larger scales. However, because requirements regarding product dimensions, pressure, cycle time, and throughput are different at an industrial scale, a similar optimization strategy to fabricate specific products might be necessary. Processing of PHBHHx on industrial scale equipment can include sheet and film extrusion, as well as thin-walled injection molding for packaging applications, using appropriate mold and chill roll temperatures.

## Figures and Tables

**Table 1:** Extrusion and injection molding parameter settings in the FFD approach.

Parameter	Value
Feeding rate (rpm)	4
Screw speed (rpm)	Variable
Processing/Cylinder temperature (°C)	Variable
Mold temperature (°C)	Variable
Cooling time (s)	Variable
Injection pressure (bar)	200
Injection time (s)	15
Post pressure (bar)	150
Post pressure time (s)	10

**Table 2:** FFD test matrix with melt processing factor symbols (**P**, **M**, **S**, **C**), low (−), middle (0), and high (+) levels.

Independent Variable	Symbol	Low (−)	Middle (0)	High (+)
Processing temperature (°C)	**P**	145	152.5	160
Mold temperature (°C)	**M**	40	60	80
Screw speed (rpm)	**S**	50	100	150
Cooling time (s)	**C**	60	180	300

**Table 3:** Extrusion temperature profiles controlling nine temperature zones: seven extruder barrel zones (Z), one die temperature (D), and one feeder temperature (F).

F ^1^	Z1	Z2	Z3	Z4	Z5	Z6	Z7	D
RT	140	140	140	140	145	145	145	145
RT	140	140	145	145	150	150	153	153
RT	140	140	150	150	155	155	160	160

^1^ Feeder temperature is approximately 23 °C, no conditioning system was used for temperature and RH control.

**Table 4:** FFD with factors, levels, and response data (mechanical properties) for tensile strength, elongation at break, and Young’s modulus (*n* = 3, ±1 Standard Deviation (SD)).

Nr	P	M	S	C	TS (MPa)	ε (%)	E (MPa)
1	−	−	−	−	21.6 ± 0.4	103 ± 15	1014 ± 25
2	−	−	−	+	22.4 ± 0.1	113 ± 16	1084 ± 8
3	−	−	+	−	21.4 ± 0.6	194 ± 46	1026 ± 14
4	−	−	+	+	21.5 ± 0.5	143 ± 19	1043 ± 15
5	−	+	−	−	21.5 ± 0.5	366 ± 9	965 ± 26
6	−	+	−	+	21.5 ± 0.2	360 ± 22	906 ± 14
7	−	+	+	−	21.2 ± 0.6	346 ± 27	951 ± 31
8	−	+	+	+	21.3 ± 0.0	321 ± 66	947 ± 17
9	+	−	−	−	20.8 ± 0.7	143 ± 39	1014 ± 30
10	+	−	−	+	21.3 ± 0.2	183 ± 7	1021 ± 8
11	+	−	+	−	21.0 ± 0.3	141 ± 24	1011 ± 18
12	+	−	+	+	21.4 ± 0.4	189 ± 63	1031 ± 13
13	+	+	−	−	20.9 ± 0.4	285 ± 59	918 ± 38
14	+	+	−	+	20.8 ± 0.8	272 ± 59	919 ± 13
15	+	+	+	−	20.6 ± 0.1	297 ± 82	872 ± 27
16	+	+	+	+	20.9 ± 0.3	358 ± 23	889 ± 54
17	0	0	0	0	20.9 ± 0.3	318 ± 78	971 ± 5

**Table 5:** Significant terms, estimates, and regression details of the tensile strength (TS) model.

Model Term *	Parameter Estimate	Standard Error	t-Ratio	*p*-Value
Intercept (MPa)	21.24	0.07	311.99	<0.0001
**P** (°C)	−0.29	0.07	−4.19	0.0009
**M** (°C)	−0.17	0.07	−2.41	0.0306

* Model terms S, C, P × M, P × S, M × S, P × C, M × C and S × C show a *p*-value > 0.05. Therefore, they are excluded from the model development and not shown in the table.

**Table 6:** Significant terms, estimates, and regression details of the elongation at break (ε) model.

Model Term *	Parameter Estimate	Standard Error	t-Ratio	*p*-Value
Intercept (%)	243.06	9.68	25.11	<0.0001
**M** (°C)	87.25	9.98	8.74	<0.0001

* Model terms P, S, C, P × M, P × S, M × S, P × C, M × C and S × C show a *p*-value > 0.05. Therefore, they are excluded from the model development and not shown in the table.

**Table 7:** Significant terms, estimates, and regression details of Young’s modulus (E) model.

Model Term *	Parameter Estimate	Standard Error	t-Ratio	*p*-Value
Intercept (MPa)	975.41	5.42	179.96	<0.0001
**P** (°C)	−16.31	5.59	−2.92	0.0112
**M** (°C)	−54.81	5.59	−9.81	<0.0001

* Model terms S, C, P × M, P × S, M × S, P × C, M × C and S × C show a *p*-value > 0.05. Therefore, they were excluded from the model development and not shown in the table.

**Table 8:** Molecular weight details (M_w_ and M_n_) of PHBHHx pellets and samples produced at **P** = 145 °C, 160 °C, 170 °C, and 180 °C determined with GPC (SEC).

Sample	M_w_ (g/mol)	M_n_ (g/mol)
Neat pellet	3.1 × 10^5^	1.0 × 10^5^
145 °C	2.7 × 10^5^	1.1 × 10^5^
160 °C	2.4 × 10^5^	9.7 × 10^4^
170 °C	2.5 × 10^5^	9.2 × 10^4^
180 °C	1.6 × 10^5^	6.3 × 10^4^

**Table 9:** Validation of TS, ε and E of PHBHHx samples produced using 2 different processing conditions: (A) **P** = 145 °C, **M** = 80 °C, **S** = 50 rpm, **C** = 60 s; (B) **P** = 160 °C, **M** = 40 °C, **S** = 50 rpm, **C** = 60 s (*n* = 5, α = 0.05).

	Tensile Speed (mm/min)	Tensile Strength (MPa)		Elongation at Break (%)		Young’s Modulus (MPa)	
		Predicted	CI Observed	Predicted	CI Observed	Predicted	CI Observed
**A**	20	21.4	21.4–22.0	330	320–342	937	883–1007
	1	/	20.5–20.7	/	135–276	/	972–995
**B**	20	21.1	21.3–22.7	156	153–325	1014	940–1205
	1	/	20.0–20.1	/	19–93	/	1011–1027

**Table 10:** Melting peak enthalpy (ΔH_m_ = ΔH_m,I_ + ΔH_m,II_ + ΔH_m,III_), endothermal peak temperatures (T_I_, T_II_, and T_III_) and percentage crystallinity (X_c_) determined with DSC at 20 °C/min for mold temperatures **M** ranging from 40 °C to 80 °C (*n* = 2, ±1 SD).

Mold Temperature (°C)	_m_ (J/g)	T_I_ (°C)	T_II_ (°C)	T_III_ (°C)	X_c_ (%)
40	43.8 ± 1.2	86.4 ± 0.1	107.4 ± 0.5	125.9 ± 0.0	38.1 ± 1.0
50	44.1 ± 2.5	87.7 ± 1.6	109.9 ± 0.7	126.6 ± 0.3	38.4 ± 2.1
60	43.8 ± 2.4	88.8 ± 0.1	112.4 ± 0.0	127.6 ± 0.0	38.1 ± 2.1
70	45.3 ± 0.4	84.2 ± 0.0	115.6 ± 0.1	129.5 ± 0.5	39.4 ± 0.3
80	44.2 ± 0.7	84.9 ± 1.0	118.5 ± 0.6	130.9 ± 0.6	38.4 ± 0.6

**Table 11:** Overview on the influence of mold temperature on the orientation function *f*, tensile properties (TS, *ε* and E), and crystallinity content (X_c_).

Mold Temperature (°C)	*f* *	TS (MPa)	*ε* (%)	E (MPa)	X_c_ (%)
40	0.14	22.6 ± 0.4	117 ± 79	1043 ± 19	38.1 ± 1.0
50	0.13	22.3 ± 0.4	216 ± 113	1018 ± 21	38.4 ± 2.1
60	0.07	22.1 ± 0.3	306 ± 79	977 ± 30	38.1 ± 2.1
70	0.04	22.2 ± 0.3	301 ± 70	957 ± 16	39.4 ± 0.3
80	0.02	22.0 ± 0.5	318 ± 41	947 ± 48	38.4 ± 0.6

* Error of the orientation function *f* is estimated to be ± 0.02.

## Data Availability

Data is contained within the article.
